# Transition to robotic adrenalectomy: a surgeon-specific learning curve evaluation

**DOI:** 10.1007/s11701-026-03440-3

**Published:** 2026-05-15

**Authors:** Sezer Akbulut, Tugba Matlim Ozel, Aykut Celik, Gorkem Yildiz, Emrecan Deniz, Serkan Sari

**Affiliations:** Department of General Surgery, Division of Endocrine Surgery, University of Health Sciences, Basaksehir Cam and Sakura City Hospital, Istanbul, Turkey

**Keywords:** Adrenal gland surgery, Robotic adrenalectomy, Robotic surgery, Learning curve, Minimally invasive surgery

## Abstract

Robotic adrenalectomy is increasingly used in selected centers; however, surgeon-specific learning patterns remain unclear. This retrospective single-center cohort study included adult patients who underwent robotic transabdominal adrenalectomy between March 2024 and December 2025. Total operative time and surgeon-dependent console time were analyzed sequentially using LOESS smoothing and learning curve cumulative sum (LC-CUSUM) analysis with predefined thresholds. Fifty-six patients were included. Mean operative time was 137.8 ± 41.3 min, and mean console time was 91.0 ± 34.4 min. Learning curve analyses showed rapid early improvement followed by stabilization, with a plateau after approximately 32 cases. LC-CUSUM analysis indicated early proficiency for operative time at the 10th–12th case, whereas proficiency based on stricter console thresholds was achieved at approximately the 36th case. Robotic transabdominal adrenalectomy achieves early proficiency, while stabilization of surgeon-dependent console performance requires a longer learning period, supporting a multiphase learning curve model.

## Introduction

Minimally invasive techniques are now firmly established as the standard approach in adrenal gland surgery, providing clinical outcomes comparable to open adrenalectomy while reducing operative morbidity [1]. Laparoscopic adrenalectomy (LA) continues to represent the conventional standard for most adrenal tumors; however, an increasing number of specialized centers have gradually shifted toward robotic adrenalectomy (RA) [2, 3]. This transition has been driven in part by the widespread dissemination of robotic platforms, which allow surgeons to perform highly precise maneuvers within confined operative spaces, minimize physiologic tremor, enhance spatial orientation through stable high-definition three-dimensional visualization, and benefit from improved ergonomics at the surgical console [4].

Robotic technology has been increasingly utilized and reported as a feasible option in technically demanding adrenal cases, including patients with obesity, hormonally active tumors, large lesions exceeding 6 cm, and those with a history of prior abdominal surgery [5]. Consistent with this expanding role, national data from the United States indicate that robotic techniques were utilized in approximately one-third of all adrenalectomies as early as 2016 [6]. This growing utilization has been accompanied by a parallel increase in interest in RA, with more frequent integration of robotic platforms into contemporary surgical training programs [7]. Nevertheless, concerns related to cost, access to robotic systems, prolonged operative times during early adoption, and the learning curve associated with transitioning to a robotic practice have continued to limit the broader dissemination of RA [1].

The learning curve (LC) is defined as the number of procedures required for a surgeon to achieve surgical outcomes comparable to those of the established gold standard technique and is clinically relevant due to its direct impact on operative time, estimated blood loss, conversion rates, and postoperative complications. Previous studies have demonstrated a progressive reduction in operative time with increasing surgeon experience [8]. In robotic surgery, this process deserves particular attention, as the adoption of robotic platforms is associated with a substantial learning curve, even among surgeons with extensive experience in advanced laparoscopic procedures [9]. These findings indicate that prior proficiency in minimally invasive surgery does not fully obviate the need for a dedicated adaptation period when transitioning to robotic techniques.

The aim of the present study is to analyze the surgeon-specific learning curve associated with the adoption of robotic adrenalectomy in a high-volume tertiary referral center with substantial experience in both open and laparoscopic adrenal surgery. Despite this institutional expertise, the transition to robotic adrenalectomy represents the implementation of a novel surgical technique and platform. Previous studies have suggested that the learning curve required to overcome the initial increase in operative time during the transition to a robotic approach may be shortened in surgeons with prior robotic or advanced laparoscopic experience [10]. By systematically analyzing time-dependent operative and console-based parameters during this transition, the present study aims to delineate surgeon-specific learning patterns in robotic adrenalectomy and to clarify the distinction between early proficiency and subsequent performance stabilization.

## Materials and methods

This retrospective single-center cohort study was conducted at the Department of Endocrine Surgery, Basaksehir Cam and Sakura City Hospital, Istanbul, Turkey, a high-volume tertiary referral center performing approximately 40 minimally invasive adrenalectomies annually since June 2020 [2]. The institutional robotic adrenalectomy program was initiated in March 2024. All patients who underwent robotic adrenal surgery between March 2024 and December 2025 and were evaluated by a multidisciplinary team comprising endocrinologists, radiologists, and endocrine surgeons were screened. Initially, 65 patients were identified. Patients aged ≥ 18 years who underwent robotic transabdominal adrenalectomy were considered eligible for inclusion.

Patients who underwent robotic retroperitoneoscopic adrenalectomy (*n* = 1) were excluded. Additionally, cases that required conversion to open surgery (*n* = 2) or laparoscopic surgery (*n* = 2) following an initial robotic approach were excluded from the analysis. All procedures were routinely video-recorded, and operative videos were reviewed individually; patients with non-analyzable or unavailable video recordings were excluded (*n* = 2). To ensure consistency in surgical technique and learning curve assessment, procedures performed by surgeons other than the primary operating surgeon were excluded (*n* = 1). Furthermore, patients with incomplete or missing data were not included in the final analysis (*n* = 1). After applying these exclusion criteria, a total of 56 patients constituted the final study cohort.

Baseline demographic and clinical variables, including age, sex, body mass index (BMI), American Society of Anesthesiologists (ASA) score, history of previous abdominal surgery, and preoperative diagnosis, were collected and analyzed using the institutional electronic database. Following detailed review of operative video recordings, intraoperative variables such as operative time, console time, conversion to laparoscopic or open surgery, and intraoperative complications were recorded. Postoperative outcomes included length of hospital stay, requirement for blood transfusion, postoperative complications, pathological findings, and 30-day mortality. The study protocol was approved by the Ethics Committee of Basaksehir Cam and Sakura City Hospital, and the requirement for written informed consent was waived due to the retrospective nature of the study.

### Surgical procedure

#### Robotic transabdominal approach

Robotic lateral transabdominal adrenalectomy was performed using the da Vinci^®^ Xi Robotic Surgical System (Intuitive Surgical Inc., Sunnyvale, CA, USA). Patients were positioned in the lateral decubitus position, with table flexion applied to enhance surgical exposure by increasing the distance between the costal margin and the iliac crest. Abdominal access was achieved using an optical entry technique, and the camera port was placed approximately 2 cm below the costal margin along the anterior axillary line. For right-sided procedures, three additional robotic trocars were placed along the subcostal line, together with an assistant port. In left-sided adrenalectomy, two robotic trocars were inserted following placement of the camera port, and an assistant port was positioned in the ipsilateral lower quadrant [11]. A Maryland bipolar forceps was introduced through the medial port, while monopolar scissors were placed through the lateral ports. These instruments were used consistently for both dissection and hemostasis throughout the procedure, without the use of any additional vessel-sealing devices. The adrenal vein was identified under direct visualization and secured using either Hem-o-lok or metallic clips before completion of gland mobilization.

### Operative standardization

All procedures were routinely video-recorded, and all operative videos were independently reviewed and analyzed by a single reviewer (S.A.). To ensure technical consistency and minimize inter-operator variability, all robotic adrenalectomies were performed by a senior endocrine surgeon (S.S.) with more than 15 years of experience in minimally invasive adrenal surgery, assisted by a European board-certified general surgeon (T.M.O.) specialized in endocrine surgery. Both the primary console surgeon and the assistant surgeon had successfully completed robotic surgery training and certification according to the Intuitive certification pathway prior to the initiation of the robotic adrenalectomy program. Since robotic surgery has been routinely performed at our institution since 2020, all procedures were carried out in a dedicated robotic operating room with a surgical team experienced in robotic procedures. Within this standardized operative setting, learning curve assessment was designed to focus on surgeon-specific performance. Accordingly, console time was selected as the primary parameter to evaluate the surgeon’s learning curve, while setup and docking times were deliberately excluded to avoid confounding effects related to operating room logistics and team experience. Console time was determined through individual review of recorded operative videos for each case. Total operative time was defined as the interval from skin incision to skin closure. All patients received perioperative antibiotic and antithrombotic prophylaxis according to institutional protocols, and surgical drains were routinely placed.

### Statistical analysis

Statistical analyses were performed using SPSS Statistics software (Version 25.0, IBM Corp., Armonk, NY, USA). Graphs and figures related to learning curve analyses were generated using R Studio software (Version 4.2.2, R Foundation for Statistical Computing, Vienna, Austria). Descriptive statistics for categorical variables were presented as number (n) and percentage (%). Comparisons of proportions between categorical variables were conducted using the chi-square test or Fisher’s exact test, as appropriate. The distributional properties of continuous variables were assessed using the Kolmogorov–Smirnov test; additionally, the assumption of normality was visually examined using histograms and Q–Q plots. Homogeneity of variances was evaluated using Levene’s test. For comparisons between two groups, Student’s t-test was used for continuous variables with normal distribution, and results were presented as mean ± standard deviation (SD). For continuous variables without normal distribution, the Mann–Whitney U test was applied, and data were reported as median (minimum–maximum). In all statistical analyses, a *p* value < 0.05 was considered statistically significant. To identify factors affecting total operative time, univariate linear regression analyses were initially performed; variables found to be significant at the *p* < 0.10 level in univariate analyses were subsequently included in the multivariate linear regression model, taking clinical relevance into consideration.

### Learning curve and LC-CUSUM analysis

Learning curve assessment was performed separately for total operative time and console time on the basis of consecutive cases. For each case, operative times were arranged according to chronological case order and presented as raw learning curve plots. To more clearly demonstrate temporal trends, 7-case moving average and LOESS (locally estimated scatterplot smoothing) methods were applied to the data to generate trend lines. The 7-case moving average was selected because it provides a balanced level of smoothing that reduces short-term variability while preserving the overall trend and represents approximately 12.5% of the total series. In addition, segmented linear regression analysis was performed to statistically identify potential breakpoints (change points) in the learning curve, and significant changes in slope according to case sequence were tested.

To determine the timing of surgical proficiency acquisition more objectively, Learning Curve Cumulative Sum (LC-CUSUM) analysis was applied. Rather than relying on descriptive reductions in operative time, LC-CUSUM analysis enables identification of the case number at which surgical performance reaches a statistically reliable level of proficiency based on predefined success thresholds and decision limits (h). In the LC-CUSUM analysis, each case was classified as “successful” or “unsuccessful” according to predefined time thresholds. Success criteria for total operative time were evaluated separately using two different thresholds, ≤ 150 min and ≤ 180 min [10]. For console time, thresholds of ≤ 90 min and ≤ 120 min were used, taking into account data distribution and clinical applicability. Analyses were repeated independently for each threshold value. The LC-CUSUM statistic was calculated cumulatively across consecutive cases. In this calculation, a weighted updating approach was used for success and failure outcomes; cases below the threshold were defined to shift the LC-CUSUM score downward, whereas cases above the threshold shifted it upward. In the analysis, the unacceptable failure rate (p₀) and acceptable failure rate (p₁) parameters were set at p₀ = 0.35 and p₁ = 0.20, respectively [adequate performance (p₁) was defined as 20% of cases exceeding the predefined operative time threshold (i.e., 80% of cases completed within the threshold), whereas inadequate performance (p₀) was defined as 35% of cases exceeding the predefined threshold]. These values were selected to be consistent with parameters used in previously published learning curve studies on robotic adrenalectomy [10, 12]. The point of surgical proficiency acquisition was defined as the first downward crossing of the predefined decision limit (h) by the LC-CUSUM curve. In accordance with the literature, the decision limit was set at h = − 1.5. Accordingly, for each metric (total operative time and console time) and each threshold value, the case number at which proficiency was achieved was reported as the case at which the LC-CUSUM curve first crossed the h = − 1.5 boundary [10, 12]. The selection of p₀ and p₁ parameters was based on previously published LC-CUSUM applications in robotic adrenalectomy, reflecting clinically meaningful thresholds distinguishing acceptable from unacceptable surgical performance. Similarly, the decision limit (h = − 1.5) was adopted from prior studies to ensure methodological consistency and comparability across learning curve analyses.

## Results

A total of 56 patients who underwent robotic transabdominal adrenalectomy were included in the study (Table 1). The mean age of the patients was 50.88 ± 11.52 years, and the mean body mass index (BMI) was 31.52 ± 6.13 kg/m². The mean operative time was 137.79 ± 41.25 min, the mean console time was 91.02 ± 34.39 min, the mean length of hospital stay was 2.88 ± 1.38 days, and the mean follow-up duration was 6.48 ± 5.51 months. The mean diameter of the pathological lesions was 3.97 ± 2.23 cm. Of the patients, 67.9% (*n* = 38) were female and 32.1% (*n* = 18) were male. According to the preoperative ASA classification, 50.0% (*n* = 28) of the cases were ASA II, 48.2% (*n* = 27) were ASA III, and 1.8% (*n* = 1) were ASA I. The vast majority of patients 98.2% (*n* = 55) had no history of previous surgery, while only 1.8% (*n* = 1) had a prior surgical history. Perioperative blood transfusion was required in 5.4% (*n* = 3) of cases.

**Table 1:** Patient characteristics of robotic transabdominal adrenalectomy (*n* = 56)

Variable	Groups	*n* (%) or Mean ± SD (Range)
Sex	Female	38 (67.9%)
	Male	18 (32.1%)
Age (years)		50.88 ± 11.52 (21–72)
Body mass index (kg/m²)		31.52 ± 6.13 (23.1–56.3)
ASA score	I	1 (1.8%)
	II	28 (50%)
	III	27 (48.2%)
Previous surgery	No	55 (98.2%)
	Yes	1 (1.8%)
Perioperative blood transfusion	No	53 (94.6%)
	Yes	3 (5.4%)
Operative time (min)		137.79 ± 41.25 (85–295)
Console time (min)		91.02 ± 34.39 (44–230)
Length of hospital stay (days)		2.88 ± 1.38 (1–8)
Follow-up duration (months)		6.48 ± 5.51 (1–18)
Tumor size (cm)		3.97 ± 2.23 (0.4–12.0)
Laterality	Left	34 (60.7%)
	Right	22 (39.3%)
Clip type	Metal clip	51 (91.1%)
	Hem-o-lok clip	5 (8.9%)
Indication	Cushing syndrome	26 (46.4%)
	Conn syndrome	12 (21.4%)
	Non-functioning adenoma	10 (17.9%)
	Pheochromocytoma	6 (10.7%)
	Metastasis	1 (1.8%)
	Adrenocortical carcinoma	1 (1.8%)
Pathological diagnosis	Adrenal cortical adenoma	43 (76.8%)
	Pheochromocytoma	4 (7.1%)
	Adrenocortical carcinoma	3 (5.4%)
	Metastasis	1 (1.8%)
	Myelolipoma	2 (3.6%)
	Lymphangioma	2 (3.6%)
	Schwannoma	1 (1.8%)
Postoperative complications	None	54 (96.4%)
	Postoperative	2 (3.6%)
30-day mortality	Yes	0 (0%)
	No	56 (100%)

All patients were operated on using a robotic surgical approach. Among patients in whom clips were applied, metal clips were used in 91.1% (*n* = 51) and Hem-o-lok clips in 8.9% (*n* = 5). Evaluation of preoperative diagnoses revealed that the most common indication was Cushing syndrome (46.4%, *n* = 26), followed by Conn syndrome (21.4%, *n* = 12), nonfunctioning adenoma (17.9%, *n* = 10), pheochromocytoma (10.7%, *n* = 6), metastasis (1.8%, *n* = 1), and adrenocortical carcinoma (1.8%, *n* = 1). Regarding lesion localization, 60.7% (*n* = 34) of cases were located in the left adrenal gland and 39.3% (*n* = 22) in the right adrenal gland.

On pathological examination, the most frequent diagnosis was adrenocortical adenoma (76.8%, *n* = 43), followed by pheochromocytoma (7.1%, *n* = 4) and adrenocortical carcinoma (5.4%, *n* = 3). Metastasis (1.8%, *n* = 1), myelolipoma (3.6%, *n* = 2), lymphangioma (3.6%, *n* = 2), and schwannoma (1.8%, *n* = 1) were among the less frequently observed pathologies (Table 1).

In the postoperative period, 96.4% (*n* = 54) of patients had no postoperative complications, whereas postoperative complications occurred in 3.6% (*n* = 2). The postoperative complications included one incarcerated incisional hernia and one patient who required re-exploration due to a postoperative hematoma. All intraoperative complications resulted in conversion to laparoscopic or open surgery and were therefore excluded from the analysis. Four conversion cases occurred at cases 8, 23, 27, and 62. Two cases (23 and 27) required conversion to open surgery due to massive bleeding and tumor adherence requiring vascular control, respectively. Two cases were converted to laparoscopic approaches: one to a retroperitoneal laparoscopic approach due to limited exposure caused by a markedly enlarged fatty liver (case 8), and one to a transabdominal laparoscopic approach due to a technical malfunction of the robotic arm used for liver retraction (case 62). There was no 30-day postoperative mortality.

No statistically significant association was found between sex and lesion localization (right/left adrenal) (*p* = 0.225). Similarly, no significant differences were observed between sex and pathological diagnosis distribution (*p* = 0.101), ASA classification (*p* = 0.710), or preoperative diagnosis groups (*p* = 0.525). There were no significant differences between female and male patients in terms of age (50.34 ± 10.90 vs. 52.00 ± 13.01 years, *p* = 0.620) or BMI (32.00 ± 6.86 vs. 30.49 ± 4.18 kg/m², *p* = 0.393). In contrast, the pathological lesion diameter was significantly larger in male patients compared with female patients (5.47 ± 2.69 cm vs. 3.26 ± 1.56 cm, *p* = 0.004). The median total operative time was also significantly longer in male patients than in female patients [135 (107–295) min vs. 122.5 (85–290) min, *p* = 0.029]. Although console time tended to be longer in male patients, this difference did not reach statistical significance [93.5 (65–230) min vs. 76.5 (44–211) min, *p* = 0.054].

**Fig. 1:**
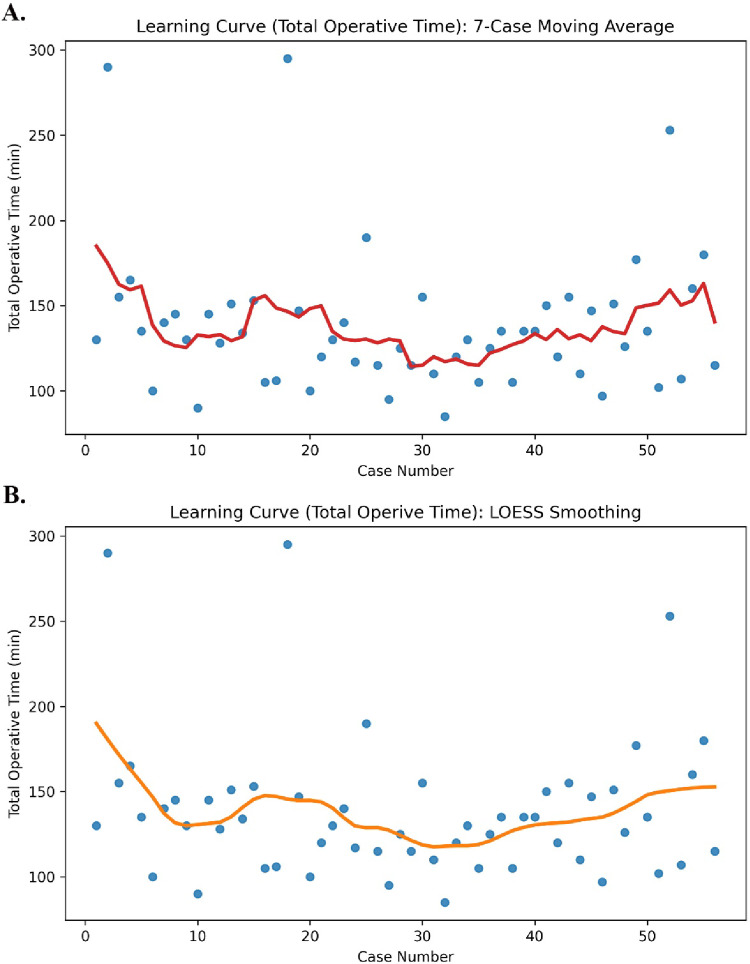
Learning curve of total operative time. (**A**) Raw operative times with 7-case moving average. (**B**) LOESS smoothing curve demonstrating the overall trend. Each dot represents an individual case, and solid lines represent smoothed trends

**Fig. 2:**
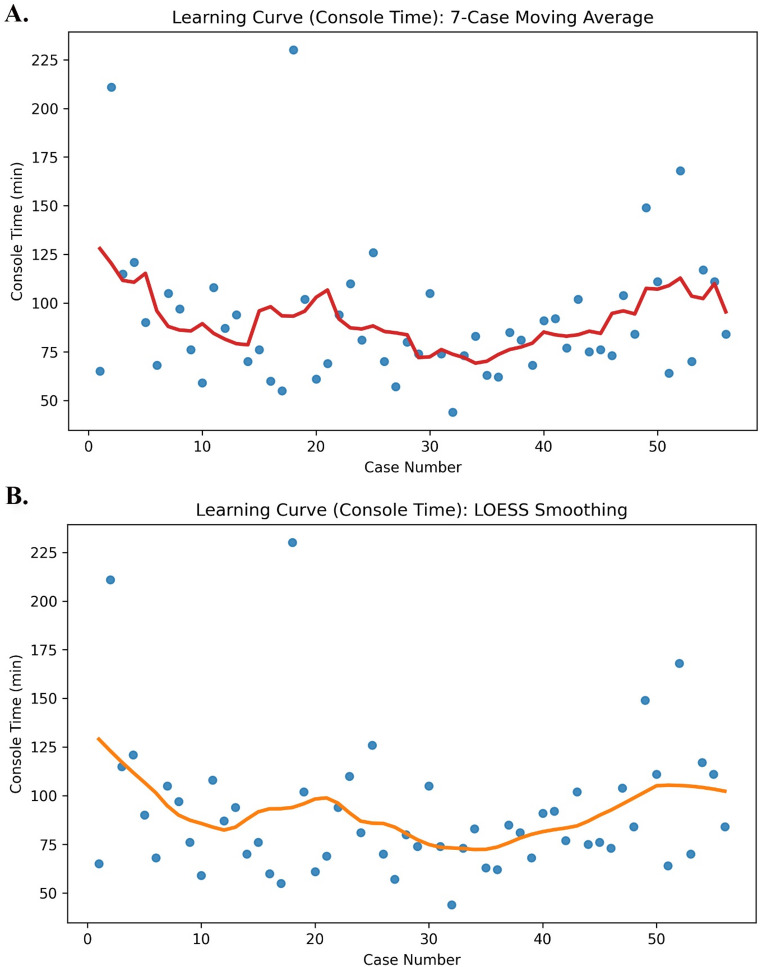
Learning curve of console time. (**A**) Raw console times with 7-case moving average. (**B**) LOESS smoothing curve demonstrating the overall trend. Each dot represents an individual case, and solid lines represent smoothed trends

Learning curves for total operative time and console time were analysed based on 56 consecutive cases. In the raw learning curve plots, a marked decreasing trend was observed in both parameters during the early phase as the case number increased. Trend lines generated using a 7-case moving average demonstrated pronounced fluctuations in operative times in the early period, followed by greater stabilization in later cases (Figs. 1A and 2A). Similarly, curves obtained using LOESS smoothing confirmed a rapid improvement during the initial learning phase, followed by a transition to a plateau phase (Figs. 1B and 2B). Joint evaluation of the LOESS and 7-case moving average curves revealed that, for both parameters, an initial phase of substantial improvement was followed by a second optimization phase, after which the slope of the curve decreased markedly and assumed a relatively horizontal course after approximately the 32nd case. Segmented linear regression analysis supported these visual findings, demonstrating a significant decreasing trend in total operative time during the first 32 cases (approximately − 1.40 min per case), whereas after the 32nd case, the slope changed significantly and no further decreasing trend in operative time was observed (*p* = 0.034 for slope change). This plateau/stabilization phase was particularly characterized by narrower variability in operative times between cases 32 and 45 and the absence of a pronounced downward slope (Figs. 1A–B and 2A–B). A plateau phase was identified after approximately the 32nd case, variability in operative and console times persisted during this phase, indicating relative stabilization rather than complete elimination of performance fluctuations.

**Fig. 3:**
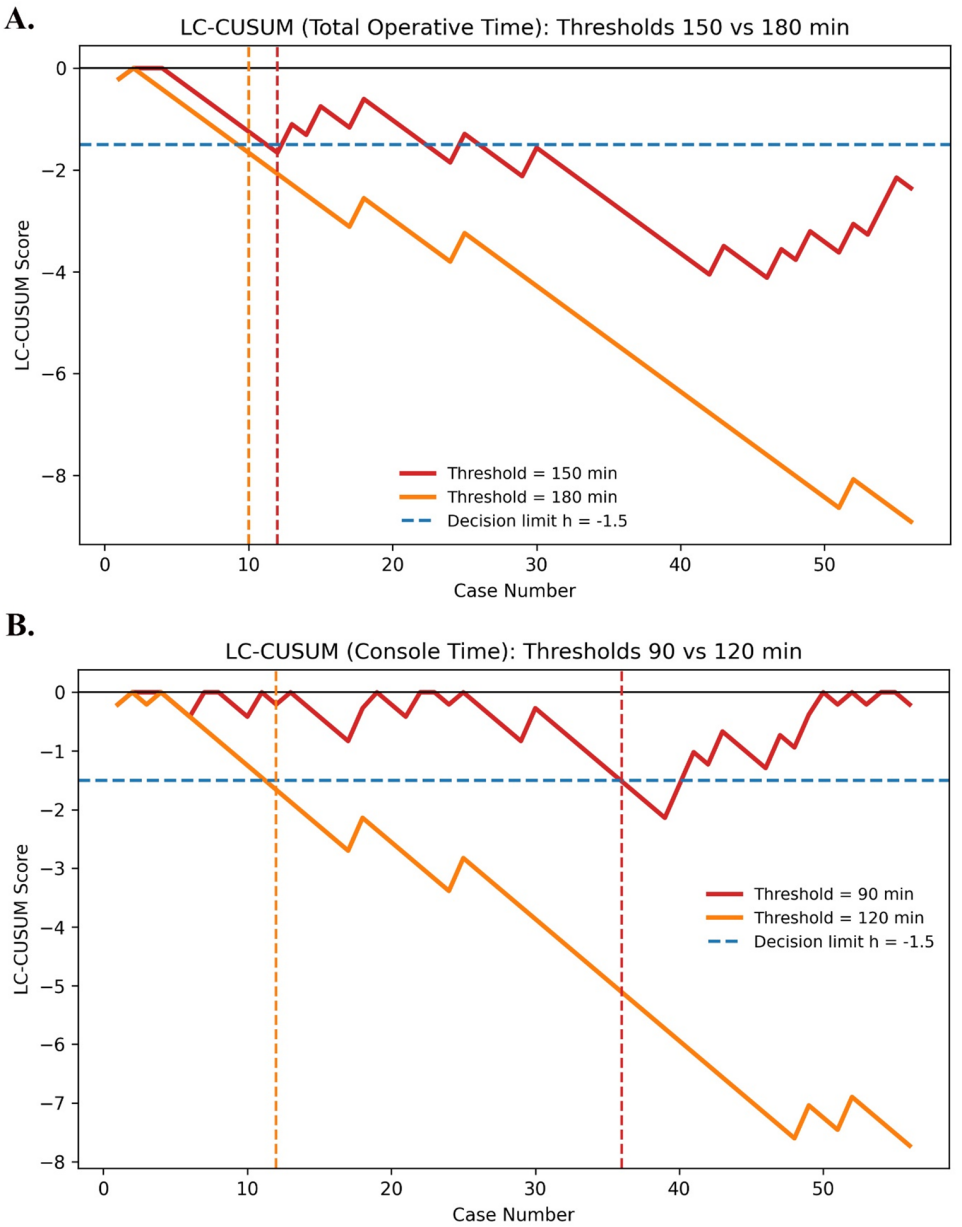
LC-CUSUM analysis of operative and console times. (**A**) LC-CUSUM analysis of total operative time using thresholds of ≤ 150 and ≤ 180 min. (**B**) LC-CUSUM analysis of console time using thresholds of ≤ 90 and ≤ 120 min. The horizontal dashed line indicates the decision limit (h = − 1.5), and vertical dashed lines represent the point at which the LC-CUSUM curve crosses this limit, indicating attainment of surgical proficiency

LC-CUSUM analysis for total operative time was performed using two different success thresholds. When a threshold of ≤ 180 min was applied, the LC-CUSUM curve crossed the decision limit of h = − 1.5 downward at the 10th case, indicating attainment of surgical proficiency at this point. Using the more stringent threshold of ≤ 150 min, the proficiency point was identified at the 12th case (Fig. 3.A). For console time, LC-CUSUM analysis was conducted using two thresholds, ≤ 120 min and ≤ 90 min, based on data distribution and clinical applicability. For the ≤ 120-minute threshold, the LC-CUSUM curve crossed the decision limit at the 12th case, indicating proficiency acquisition, whereas for the stricter ≤ 90-minute threshold, proficiency was achieved at the 36th case (Fig. 3.B).

Linear regression analyses were performed to identify factors affecting total operative time (Table 2). In univariate analyses, tumor size was positively associated with operative time (β unstandardized coefficient = 6.5, *p* = 0.008), and ASA III status (compared with ASA I/II) was also associated with longer operative time (B = 30.2, *p* = 0.005). The effect of sex (male vs. female) on operative time was of borderline statistical significance (B = 22.90, *p* = 0.051). No significant associations were observed between operative time and age, BMI, laterality, pathological diagnosis (others vs. adenoma), indication (functional vs. non-functional), or procedural phases (1–9 vs. 10–32; 33–56 vs. 10–32) (all *p* > 0.05). In the multivariate model, tumor size and ASA class remained independently associated with operative time; each 1-cm increase in tumor size was associated with an average increase of 5.03 min in operative time (95% CI [0.32, 9.75], *p* = 0.037). ASA III status was associated with an average increase of 24.30 min in operative time (95% CI [3.44, 45.10], *p* = 0.023). Sex did not retain statistical significance in the multivariate model (*p* = 0.339).

**Table 2:** Univariate and multivariate linear regression demonstrating factors associated total operative time in robotic adrenalectomy

Variables	Univariate			Multivariate		
	β (Coefficient)	95% CI	*P* value	β (Coefficient)	95% CI	*P* value
Age (years)	–	–	0.466			ni
BMI (kg/m²)	–	–	0.449			ni
Tumor size (cm)	6.5	1.77 & 11.2	**0.008**	5.03	0.32 & 9.75	**0.037**
Gender (male vs. female)	22.9	-0.13 & 45.9	**0.051**	–	–	0.339
ASA (3 vs. 1/2)	30.2	9.5 & 51	**0.005**	24.3	3.44 & 45.1	**0.023**
Laterality (right vs. left)	–	–	0.686			ni
Pathological diagnosis (others vs. adrenal cortical adenoma)	–	–	0.167			ni
Indication (functional vs. non-functional)	–	–	0.812			ni
Procedure number						
Procedure number (1–9 vs. 10–32)	–	–	0.556			ni
Procedure number (33–56 vs. 10–32)	–	–	1.000			ni

CI: Confidence Interval, BMI: Body Mass Index, ni: Not included β indicates the unstandardized regression coefficient

## Discussion

The adoption of robotic technology has established robotic adrenalectomy as a viable option in selected medical centers [13]. Its safety and effectiveness have been largely demonstrated using the da Vinci system, which has consequently become the reference platform for robotic surgery in clinical practice [14]. However, the evaluation of newly adopted surgical techniques remains challenging, as outcomes are influenced by multiple factors, including surgeon-related variables, team experience, learning curves, and technical variability [15]. Among these factors, the learning curve is particularly important, as it directly affects operative time, conversion rates, blood loss, and postoperative outcomes, with increasing experience consistently associated with reductions in operative time [8].

Operative time is the most commonly used parameter for evaluating surgical efficiency and learning curves in robotic adrenalectomy. In the literature, reported operative times for RA vary widely, generally ranging from 80 to 230 min, with most studies demonstrating a progressive reduction in operative time as robotic experience increases. Console time is typically reported to average between 90 and 120 min, while docking time in experienced centers is usually less than 15 min [16, 17]. In our cohort, the mean operative time was 137.79 ± 41.25 min (range, 85–295), and the mean console time was 91.02 ± 34.39 min (range, 44–230), findings that are consistent with the ranges reported in the literature. Console time represents a major contributor to prolonged operative duration during the early phase of adopting a robotic surgical practice [18]. In this context, our study aimed to characterize the learning curve of robotic transabdominal adrenalectomy during the transition to a new robotic platform in a high-volume center, focusing primarily on operative time and console-based performance metrics.

The learning curve of robotic adrenalectomy appears to be shorter than previously assumed, with surgical proficiency—commonly defined by consistent operative times, reduced complication rates, and minimal need for conversion—often achieved within 20–30 cases [19]. In line with this, Brunaud et al. reported that approximately 20 cases were sufficient to reach proficiency, whereas D’Annibale et al. described an even shorter learning curve, with proficiency attained after 12 cases [20, 21]. In our series of 56 cases, LC-CUSUM analysis demonstrated early attainment of surgical proficiency based on total operative time, with proficiency achieved at 10–12 cases depending on the selected threshold. However, analysis of surgeon-dependent console performance revealed a more gradual learning process. Time-series analyses using LOESS smoothing and segmented regression identified an initial phase of rapid improvement followed by a stabilization phase, with a clear plateau emerging after approximately 32 cases. These findings are consistent with a large multicenter study including four surgeons, in which robotic adrenalectomy learning curves were evaluated using LC-CUSUM across multiple operative time thresholds (120, 150, and 180 min), demonstrating proficiency acquisition between 8 and 29 cases depending on the individual surgeon [10]. Taken together, these data suggest that while surgical proficiency can be achieved relatively early, optimization and stabilization of console-based robotic performance may extend beyond the initial proficiency phase. Importantly, the plateau phase observed in our analysis should not be interpreted as complete stabilization, as variability in operative and console times persisted beyond this point, reflecting the inherent case-to-case variability of robotic adrenalectomy.

In a single-surgeon series of 33 robotic single-site adrenalectomies [22], Lee et al. evaluated the learning curve based on reductions in operative time and demonstrated a clear stabilization after approximately 20 cases. Similarly, in a 111-case robotic transperitoneal adrenalectomy series from two high-volume centers [18], Ozdemir et al. (2020) defined the first 20 cases as the learning phase and reported significant reductions in both total operative time and console time thereafter. Together, these studies suggest that the commonly adopted “first 20 cases” framework provides a practical benchmark for defining surgical proficiency. However, our findings indicate that when advanced analytical methods are applied, the learning curve of robotic adrenalectomy is better characterized as a multiphase process encompassing distinct proficiency and stabilization phases, rather than a single inflection point.

Several factors, including tumor complexity and obesity have been reported to prolong operative time in robotic adrenalectomy [23]. In our study, multivariate regression analysis identified tumor size and ASA score as independent predictors of total operative time. Each 1-cm increase in tumor diameter was associated with an approximate 5-minute increase in operative duration, consistent with previous reports demonstrating a significant correlation between tumor size and operative time [24]. In addition, operative time was, on average, 24 min longer in patients with ASA III status compared with those classified as ASA I–II. Accordingly, learning curve analyses in robotic adrenalectomy may be more appropriately interpreted when patient-related risk profiles and case complexity are taken into account, rather than being based solely on procedural sequence.

Artificial intelligence (AI) and machine learning (ML) are increasingly integrated into robotic surgery, with applications including predictive analytics for surgical planning, AI-based image segmentation, and real-time intraoperative decision support. In parallel, ML-driven training simulators are being used to assess performance and guide skill acquisition. Together, these developments reflect a paradigm shift in adrenal surgery from conventional laparoscopy toward a technology-enhanced, precision-oriented robotic approach [23]. In addition to technical advantages, robotic adrenalectomy offers clear ergonomic benefits by reducing physical strain and improving posture, wrist articulation, and hand stability, as demonstrated in ergonomic studies [25]. However, limitations such as high costs and the learning curve remain barriers to widespread adoption, particularly in low-volume centers [26]. Overcoming these challenges requires multidisciplinary collaboration to optimize robotic platforms, integrate them into clinical workflows, and establish standardized, evidence-based protocols. Structured training programs are essential to ensure safe and effective implementation of emerging technologies [27].

The main strength of this study lies in its comprehensive evaluation of the learning curve of robotic transabdominal adrenalectomy using multiple complementary analytical approaches, including LC-CUSUM, time-series analyses, and segmented regression. The use of surgeon-dependent console time enabled a more isolated assessment of technical skill acquisition by minimizing the confounding effects of operating room logistics and team experience. Furthermore, all procedures were video-recorded and systematically reviewed, ensuring data accuracy and consistency.

Several limitations should also be acknowledged. This was a retrospective, single-center study involving procedures performed by a single primary surgeon, which may limit the generalizability of the findings to other institutions or surgeons with different levels of experience. However, this design allowed for a focused evaluation of surgeon-specific learning patterns under standardized operative conditions, minimizing inter-operator variability. In this context, the observed learning curve likely reflects adaptation to robotic-specific technical skills rather than acquisition of fundamental adrenalectomy techniques. Learning curve characteristics may vary depending on prior surgical experience, and surgeons with extensive robotic experience in other specialties may demonstrate different or shorter adaptation patterns. In addition, the absence of a direct laparoscopic or open comparator limits the contextual interpretation of these findings. As this study was not designed as a comparative analysis between surgical approaches, the findings should be interpreted within the context of surgeon-specific learning rather than procedural superiority. Although the sample size is comparable to or larger than that of many previously published learning curve studies in robotic adrenalectomy, larger multicenter studies are warranted to validate these results. In addition, operative time–based metrics, while objective and widely used due to the low incidence of measurable adverse outcomes such as complications or blood loss, may not fully capture other dimensions of surgical proficiency, including intraoperative decision-making and long-term outcomes. Finally, although the LC-CUSUM method provides an objective framework for identifying proficiency thresholds, it does not explicitly account for variations in case complexity; however, defining acceptable and unacceptable failure rates allows for a degree of heterogeneity in case difficulty.

## Conclusion

This study demonstrates that surgical proficiency, as defined by predefined operative time thresholds, in robotic transabdominal adrenalectomy can be achieved relatively early, whereas stabilization of surgeon-dependent console performance occurs over a more extended learning period. These findings support a multiphase learning curve model and emphasize the importance of distinguishing early proficiency from subsequent performance stabilization when evaluating learning curves in robotic adrenalectomy.

## Data Availability

The datasets generated and/or analyzed during the current study are not publicly available owing to institutional regulations but are available from the corresponding author upon reasonable request.
